# Present-Day Tectonic Stress Evolution in Southern Yunnan Based on Focal Mechanisms

**DOI:** 10.3390/s23177406

**Published:** 2023-08-25

**Authors:** Wenjie Fan, Ye Zhu, Yingfeng Ji, Lili Feng, Weiling Zhu, Rui Qu

**Affiliations:** 1 State Key Laboratory of the Tibetan Plateau Earth System, Environment and Resources (TPESER), Institute of Tibetan Plateau Research, Chinese Academy of Sciences, Beijing 100101, China; fan_095011106@126.com (W.F.); zhuye@itpcas.ac.cn (Y.Z.); zhuweiling@itpcas.ac.cn (W.Z.); qurui@itpcas.ac.cn (R.Q.); 2 Yunnan Earthquake Agency, Kunming 650244, China; 3 University of the Chinese Academy of Sciences, Beijing 100049, China; 4 Qinghai Earthquake Agency, Xining 810001, China; ynufll@sina.com

**Keywords:** grid point test method, focal mechanism solution, stress field, stress shape ratio (*R*-value), southern Yunnan region

## Abstract

Tectonic extrusion bypassing the eastern Himalayan syntaxis results in a significant increase in regional stress instability and the associated frequent occurrence of earthquakes in southern Yunnan, China. However, the stress field, and the relationship between the focal mechanism of earthquakes and stress evolution in southern Yunnan, remain enigmatic. In this paper, using a modified grid point test method, we calculated the focal mechanism of *M*_L_ ≥ 2.5 earthquakes in southern Yunnan (22–25° N, 100–104° E) from January 2009 to June 2023. Utilizing the solutions of historical earthquake focal mechanisms, we obtained the present-day regional tectonic stress field in southern Yunnan via inversion. The results indicate complex and diverse seismic focal mechanisms, and the main types of earthquakes are strike-slip events, followed by normal fault and reverse fault events. The orientations of the maximum and minimum principal stress axes rotate in a clockwise direction from northeast to southwest. The internal stress orientation distribution of the rhombic Sichuan–Yunnan block in the study area is consistent, and the block boundary zone is the site where stress deflection occurs, and the regional tectonic stress field is influenced by the interaction among different blocks. The distribution of *R*-value in the Lamping–Simao block gradually increases from north to south, indicating that the compressive stress required for material transport becomes relatively small. Combined with the geological and tectonic background of the study area, our results suggest that the speed of block movement gradually decreases from north to south; the distribution of *R*-value in the South China block is significantly smaller than that of the interior of the Sichuan–Yunnan rhombus, and the proportion of compressive stresses is larger, indicating a stronger extrusion in this region, which may be related to the fact that the Sichuan–Yunnan rhombus is strongly resisted by the South China block in the east.

## 1. Introduction

Crustal stress variation influences the dynamic processes of plate tectonics and seismicity [1]. The fine structure of the continuous dynamic change in stress provides insights into the deep crustal tectonic evolution [2,3,4]. Numerous technical methods for solving the tectonic stress field were developed by predecessors using different data sources, such as inversion calculations with focal mechanism solution data [5,6,7,8], P-wave polarity data [9,10], and fault sliding data to obtain the regional stress field [11,12]. The focal mechanism solution of moderate and small earthquakes is a significant indicator of the stress field near the source [13]. In the area where earthquakes are frequent and the focal mechanism could be solved, the inversion of focal mechanism data to calculate the tectonic stress field provides a better explanation of the seismogenic environment and geodynamic background in the region.

**Figure 1:**
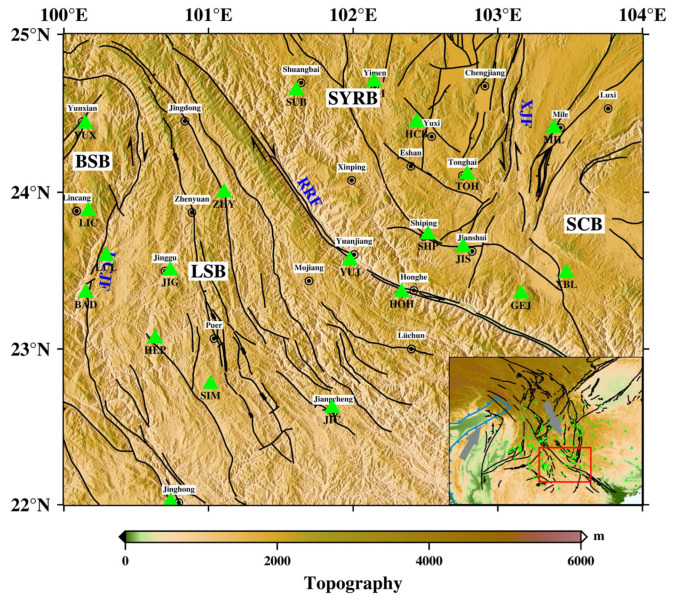
The tectonic background of southern Yunnan. The black lines denote the faults in the research area. LCJF: Lancangjiang (Lancang river) fault; RRF: Red River fault; XJF: Xiaojiang fault; SYRB: rhombic Sichuan–Yunnan block; LSB: Lamping–Simao block; BSB: Baoshan block; SCB: South China block. The green triangles are seismic stations used in the inversion. YUX: Yunxian; BAD: Bangdong; LUL: Luolian; HEP: Heping; JIG: Jinggu; LIC: Lincang; MEL: Menglian; SIM: Simao; ZHY: Zhenyuan; JIC: Jiangcheng; YUJ: Yuanjiang; HOH: Honghe; SUB: Shuangbai; SHP: Shipping; JIS: Jianshui; GEJ: Gejiu; XBL: Xibeile; MIL: Mile; HCB: Huangcaoba; YIM: Yimen; TOH: Tonghai. The inset denotes the location of the research area. The gray arrows represent the direction of plate motion. The blue line indicates the plate boundary. The red box indicates the study area.

Yunnan is located on the southeastern edge of the Tibetan Plateau, adjacent to the Himalayan East Tectonic Junction, and is one of the regions with the most complex active structures and the most frequent strong earthquake activities [14]. The collision of the Indian plate and the Eurasian plate and the northward movement of the Indian plate caused the deformation of the plate edge or internal uplift and the massif to escape to the east. During the northward movement of the Indian plate, the intersection position with the South China block is strongly blocked, causing tectonic changes such as translation, clockwise rotation, and vertical uplift of the blocks in the Sichuan–Yunnan area [15]. The study area (22–25° N, 100–104° E) spans south-central Yunnan Province, including some large fractures such as the Red River fracture zone, the Lancang River fracture, and the Xiaojiang River fracture [16]. The northern section of the Red River fault zone is boundary fracture with positive strike-slip, and the southern section contains an extrusive recoil component [15]. Since the Cenozoic, the Xiaojiang Fault has shown intense activity with the characteristics of left-slip movement, and the middle section of the fault zone has obvious characteristics of a crustal deep fracture [17]. The Nujiang–Lancang River rift zone constitutes a boundary zone of very high tectonic and strong seismic activity, which is formed by the intersection of a series of active ruptures [18]. These deep major faults divide the study area into three different tectonic blocks: the Yangzi Platform, the Baoshan block, and the Lamping–Simao block, where the Yangzi Platform is divided into the Sichuan–Yunnan rhombic block and the South China block by the Anning River–Zemu River–Xiaojiang fracture zone, and the Sichuan–Yunnan rhombic block can be further subdivided into the Northwest Sichuan and Central Yunnan subblocks ([19]; Figure 1). The mutual movement of different blocks in the study area has created stress zones at the boundaries, which are significantly influenced by block and fracture interactions, making the geological structure and mechanical properties of the region intricate and complex [20,21].

Numerous previous studies focused on the regional tectonic stress environment. For instance, the tectonic stress effects in Southwest China are found to be mainly horizontal, with the regional stress field having obvious zoning characteristics through the method of using fault slip data combined with the focal mechanism and in situ stress measurements [22]. Zhao et al. [23] calculated the tectonic stress field in Yunnan using the damped linear inversion method and suggested that the Yunnan is in a strike-slip stress regime, with the maximum and minimum principal stress directions showing clockwise rotation. [24] inferred the focal mechanism of earthquakes calculated with the cut and paste (CAP) method and recognized that the spatial distribution of different types of focal mechanism solutions varied significantly. Although the stress field direction in Yunnan shows some spatial continuity, the main stress direction varies widely in different regions. Previous studies focused on the characterization of regional tectonic stress fields at large spatial scales but did not analyze the stress shape factor *R*-value in conjunction, which may contain information on interblock interactions and stress transitions (e.g., [25]) and indicates important parameters for geodynamic models (e.g., [26]). Therefore, it is important to analyze the dynamic changes in terms of mechanical properties in which the tectonic stress can be used to explain the tectonic evolution in this region.

In this study, we used a modified grid point test method to calculate the focal mechanism solutions of *M*_L_ ≥ 2.5 seismic events in southern Yunnan that meet the requirements of P-wave polarity data and collected data to supplement the focal mechanism solutions of historical small and medium-sized earthquakes in the study area. The study area was divided into grid partitions for a fine-scale inversion of the tectonic stress field to obtain the characteristics of the tectonic stress field changes in southern Yunnan and to explore the geodynamic relationship between the tectonic stress field and the block motion in the area. This work is expected to provide insight into the relationship between the tectonic stress field and the occurrence of earthquakes, and to provide a reference basis for interpreting the earthquake dynamic processes in southern Yunnan.

## 2. Materials and Methods

### 2.1. Data

**Figure 2:**
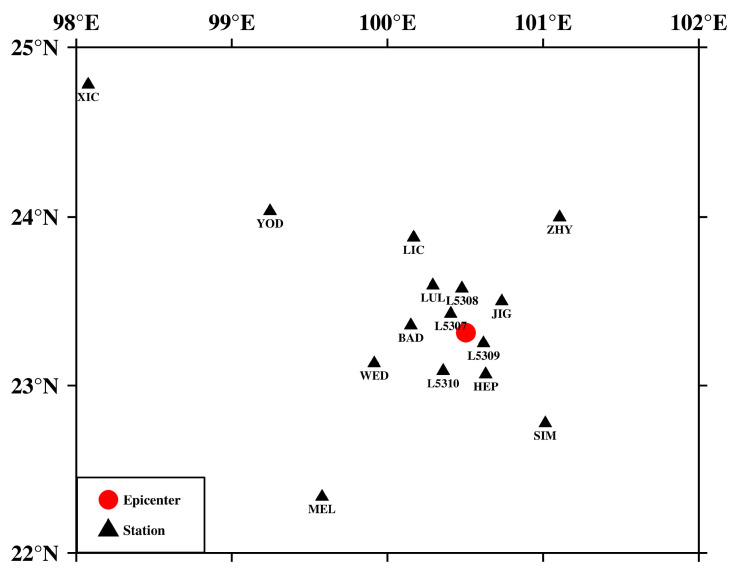
Example inversion for an *M*_L_ 2.5 event on 10 December 2014. The solid red circle is the epicenter of the earthquake, and the black triangles are the stations where the earthquake was recorded. BAD: Bundung; LUL: Luolian; WED: Wendong; XIC: Xincheng; HEP: Huaping; JIG: Jinggu; L5307: Temporary 5307; L5308: Temporary 5308; L5309: Temporary 5309; L5310: Temporary 5310; LIC: Lincang; MEL: Menglian; SIM: Simao; YOD: Yongde; ZHY: Zhenyuan.

The focal mechanism solution is crucial for understanding the source information and stress field (e.g., [27]). The physical image of the P-wave first-motion symbol is clear and contains stable seismic wave information, which has a unique advantage in determining the focal mechanism solutions. In this paper, we collected 1639 *M*_L_ ≥ 2.5 seismic events with P-wave first-motion data in southern Yunnan (22~25° N, 100~104° E) from January 2009 to June 2023. These data were from the official observation reports of the Yunnan region seismic network, and 123 stations were involved. In accordance with the limitations of seismic network stations in recording *M*_L_ ≤ 3 earthquake events in the area of Southern Yunnan and the weak sensitivity to recognize the first-motions of P-waves, only *M*_L_ ≥ 2.5 earthquakes with at least 15 symbols of the first-motions of P-waves were included in this research. We also provide an example of weak (*M*_L_ = 2.5) earthquakes obtained at different distances from the epicenter being used to determine the focal mechanism (Figure 2). The *M*_L_ = 2.5 earthquake of 10 December 2014, contains P-wave first-motion symbols recorded at 15 stations, with the furthest station (XIC) approximately 296 km and the closest station (L5309) approximately 13 km from the epicenter (Figure 2). Finally, a total of 15,211 P-wave first-motion polarity data points were obtained.

### 2.2. Calculation Method of Focal Mechanism Solutions

Xu et al. [28] proposed a grid point test method to solve the focal mechanism solutions by using the P-wave first-motion symbol and obtained the solution region (the set of alternative solutions) and average solution. However, the method still had great uncertainty when the actual observation data were not sufficient. Moreover, the method lacked a reasonable solution quality evaluation scheme for focal mechanism solutions. In view of this situation, Yu et al. [29] improved the grid point test method [28] in several aspects. For example, they proposed a method to calculate the weight of data points that depended on the quality of P-wave first-motion data and the density of data points on the focal sphere, a method to give single or multiple possible average solutions through cluster analysis, and an evaluation scheme for the quality of focal mechanism solutions. According to the new weight calculation method, the weighted inconsistency ratio of the m-th trial solution can be expressed as

(1) $$\psi_{m}=\frac{\sum_{i=1}^{N_{m}}W_{i}}{\sum_{i=1}^{N_{T}}W_{i}}$$

where $N_{m}$ is all the inconsistency points corresponding to the *m*-th solution, $N_{T}$ is all data points, and $W_{i}$ is the weight factor of the *i*-th data point. Meanwhile, in the process of cluster analysis, the minimum rotation angle of the P, B, and T coordinate system is introduced as the parameter of the clustering threshold. There is a minimum rotation angle (*θ_mn_*_min_) between each alternative solution and the average solution, and the root mean square (RMS) of *θ*_min_ can be used to describe the dispersion degree of the solution. In this study, we used *ψ*_m_ and RMS to evaluate the quality of focal mechanism solutions.

In the concrete calculation process, we scanned the 3D parameter space of all possible solutions in steps of 5° × 5° × 5° to ensure the reliability of the calculation results. The solution with the weighted inconsistency ratio *ψ* between the minimal value *ψ*_min_ and *ψ*_min_ + 5% was chosen as the optional solution (the discrete region of the solution). The final individual earthquake focal mechanism solution was determined with cluster analysis and stability testing of the solution.

### 2.3. Inversion Method of the Tectonic Stress Field

Michael [7,30,31] proposed a linear inversion algorithm to invert the stress tensor from a nonlinear problem to a linear problem [11,32]. The method is based on two assumptions: one is that the direction of shear stress on the fault surface is parallel to the direction of fault slip, and the other is that the stress field is uniform for the selected data set. It is difficult to determine which fault plane is the true seismogenic fault for inversion calculation due to the uncertainty of the focal mechanism. In the linear inversion algorithm, one of the two nodes was randomly selected to participate in the calculation. The bootstrap resampling method was used to perform multiple sampling calculations on the data, and a certain confidence interval was given to evaluate the uncertainty of the solution and finally obtain the optimal solution. Hardebeck et al. [4] introduced damping parameters into the inversion algorithm and then developed the damping region inversion algorithm, which can suppress the difference in stress modes between adjacent elements and smooth the stress tensor obtained from inversion [33], which is more suitable for the fine inversion of large regional scales. In this method, the model length and data fitting error are controlled by setting the damping coefficient. Determining the optimal damping coefficient helps to ensure the proper complexity of the model and reduce the data fitting error. It is a least-squares solution of the equation:

(***G***^T^***G*** + *e*^2^***D***^T^***D***)***m*** = ***G***^T^***d*** (2)

where ***m*** is the model vector containing the stress tensor elements at the grid points, ***d*** is the data vector including the slip vector components of earthquakes at the corresponding grid points, and ***G*** is the data kernel matrix involving the normal vector components of all the fault planes. Matrix ***D*** is the damping matrix formed by blocks of zeros, identity matrix ***I*** and its opposite −***I***, which is used to minimize the difference in stress between adjacent subareas. *e* is the damping parameter to adjust the strength of damping.

The inversion results include the direction of the three principal stress axes, the value of the stress shape factor *R*, and its uncertainty range, where *R* = (*σ*_1_ − *σ*_2_)/(*σ*_1_ − *σ*_3_) and *σ*_1_, *σ*_2_, and *σ*_3_ represent the maximum, intermediate, and minimum principal stresses, respectively. The value of the stress shape factor *R* represents the relative magnitude of the three principal stresses. If *R* = 0.5, the eigenvalues of the stress tensor are arranged in an isometric order. If the *R*-value decreases gradually from 0.5, the eigenvalues of the intermediate stress approach the maximum principal stress eigenvalues, and the intermediate stress axis shows the properties of compressive stress. If the *R*-value increases gradually from 0.5, the intermediate stress eigenvalue gradually approaches the minimum principal stress eigenvalue, and the intermediate stress axis corresponds to the property of tensile stress [1].

## 3. Results

### 3.1. Focal Mechanism Solution

**Figure 3:**
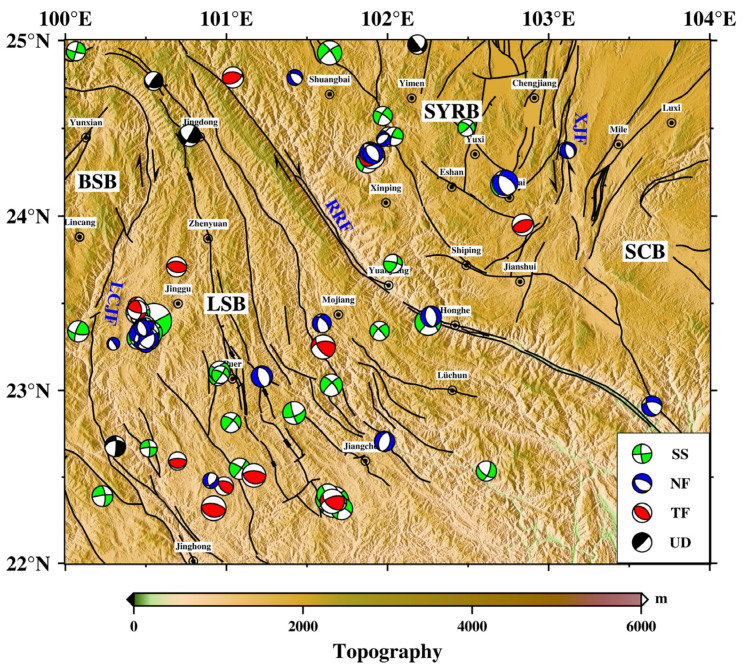
The focal mechanisms of earthquakes in southern Yunnan were calculated by using the improved procedure of Yu et al. [29] for the grid point test method [28] based on *M*_L_ ≥ 2.5 events in southern Yunnan (22–25° N, 100–104° E) from January 2009 to June 2023 involving 123 stations and a total of 15,211 P-wave first-motion polarity data.

**Figure 4:**
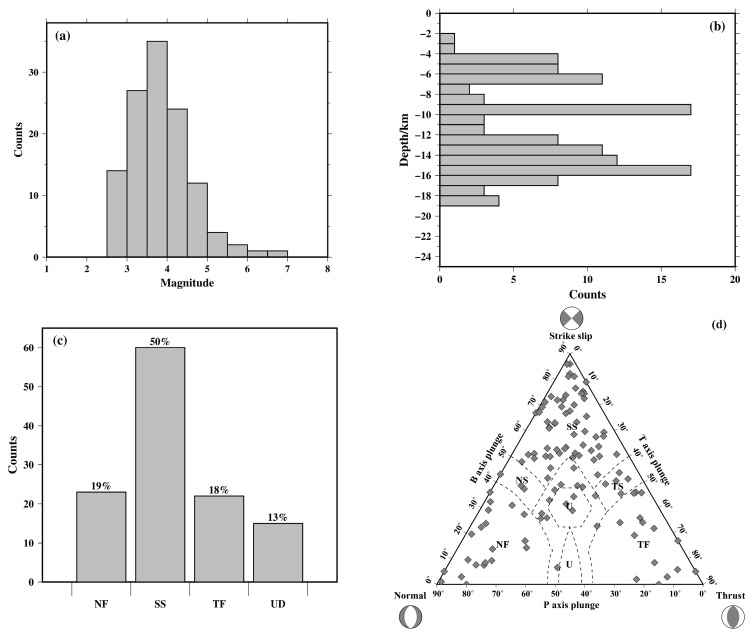
(**a**) Magnitude distribution of focal mechanism solutions from the Yunnan region earthquake catalog. (**b**) Depth distribution of focal mechanism solutions from the Yunnan region earthquake catalog. (**c**) The proportions of different types of focal mechanism solutions. NF: normal fault; SS: strike-slip; TF: thrust fault (reverse fault); UD: uncertain definition. (**d**) Ternary plot of focal mechanism data: each small square is plotted based on the plunge of the P, T, and B axes of the focal mechanism solutions [35]. The dashed lines divide the diagram into faulting styles based on definitions by Zoback [34].

According to the calculation with the improved procedure of Yu et al. [29] for the grid point test method [28], the P-wave initial polarity data of a total of 120 seismic events satisfied the calculation conditions, and we obtained the focal mechanism of earthquakes (Figure 3). The magnitudes in these solutions varied from *M*_L_ 2.5 to 6.9, with 88% of the earthquakes >*M*_L_ 3 (Figure 4a). The depth distribution of focal mechanism solutions from the Yunnan region earthquake catalog (Figure 4b) shows that these seismic events mainly occurred at depths of 5–20 km, which reflects the stress state of the upper crust to a certain extent. Referring to the world standard for the classification of stress maps [34], the focal mechanism solutions were classified into six categories based on the magnitude of the plunge in the P, B, and T axes (Table 1), including normal fault (NF), normal strike-slip fault (NS), strike-slip fault (SS), thrust strike-slip fault (TS), thrust fault (TF), and uncertain definition (UD). Based on the classification criteria, the calculated data of the focal mechanism solutions were classified and grouped into statistics. In this paper, normal faults and normal strike-slip faults are collectively referred to as normal faults, and thrust strike-slip faults and thrust faults are collectively referred to as thrust faults (reverse faults). The statistical results show that southern Yunnan is mainly dominated by strike-slip fault earthquakes, with 60 strike-slip focal mechanism solutions, accounting for 50% of all focal mechanism solutions, 22 thrust faults and 23 normal faults, accounting for 18% and 19%, respectively, and 15 uncertain earthquakes, accounting for 13% of the total (Figure 4c). To visualize the proportions of thrust, normal, and strike-slip focal mechanism solutions among the earthquakes in our dataset, we adopted the ternary diagram introduced by Frohlich [35]. This diagram provides a quick and manageable way to determine the dominant style of faulting in a particular region. Figure 4d reports the distribution of focal mechanism solutions belonging to our final dataset in the diagram. The focal mechanism solution type in the study area is mainly the strike-slip type, which is consistent with the statistical results.

**Figure 5:**
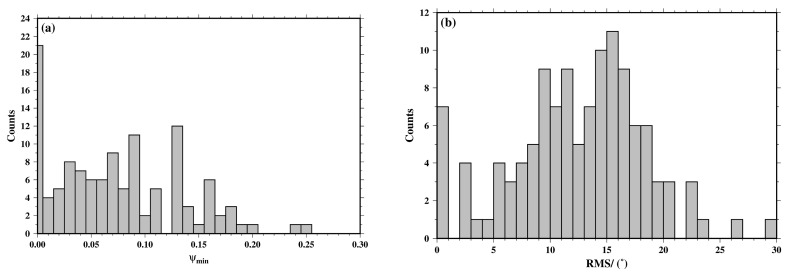
Quality evaluation of focal mechanism solutions. (**a**) Distribution histogram of the minimum weighted inconsistency ratio (*ψ*_min_). (**b**) Distribution histogram of the root mean square (RMS) of the minimum rotation angle.

To evaluate the quality of focal mechanism solutions, the distribution histogram of the minimum weighted inconsistency ratio (*ψ*_min_) and root mean square (RMS) of the minimum rotation angle are presented in Figure 5. The results show that the distributions of *ψ*_min_ and RMS are smooth and realistic. Almost 88% of *ψ*_min_ values are less than 0.15, which is generally considered accurate and reliable [28,36] (Figure 5a). As shown in Figure 5b, the RMS is also relatively low, and approximately 93% of them are below 20°, which verifies the feasibility of solving the focal mechanism with P-wave first-motion data and the reliability of the results presented in this paper.

**Figure 6:**
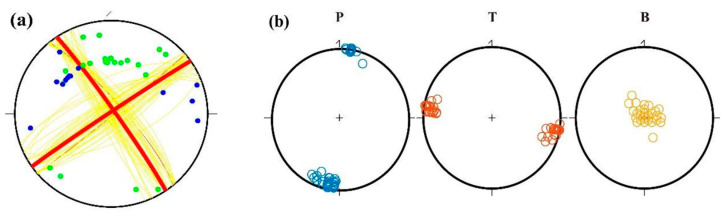
Example inversion for an *M*_L_ 4.6 event on 13 November 2015. (**a**) The lower-hemisphere equal-area projection of the focal mechanism; blue points are compressional first motions, green points are dilatational first motions, yellow lines are nodal planes of the selected solutions, and red lines are the mean solution. (**b**) P, T, and B axes of the focal mechanism solution (blue, red, and yellow circles, respectively).

An example of a successful inversion is presented in Figure 6 for an *M*_L_ 4.6 earthquake that occurred on 13 November 2015 (Figure 6). In Figure 6a, the 35 P-wave first-motion data points were evenly distributed on the focal sphere projection map, and cluster analysis showed that there was only one set of solutions. The discrete regions of the solutions of the three principal stress axes were also relatively concentrated (Figure 6b). For example, the minimum weighted inconsistency ratio (*ψ*_min_) was 0.05 (less than 0.15), and the root mean square (RMS) of the minimum rotation angle was 14.2°.

**Figure 7:**
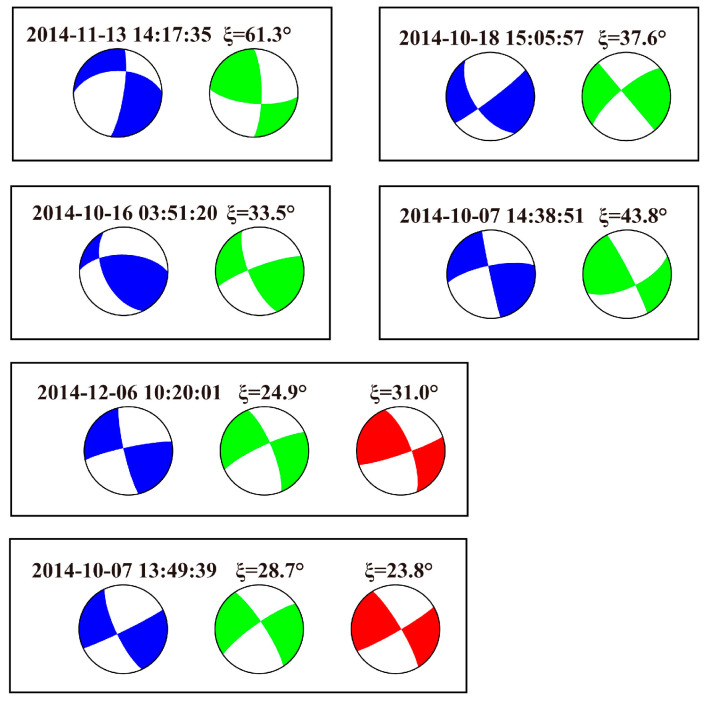
Comparison of focal mechanism solutions presented in this study and those in Xu et al. [37] or the Globe CMT.

In general, our focal mechanism results are consistent with previous research results developed by other researchers. The comparison between the six focal mechanisms presented in this study and those in Xu et al. [37] or Globe CMT is shown in Figure 7. The range of the minimum 3D rotation angle between focal mechanisms, known as Kagan’s angle [38,39], is 23.8~61.3°. We attribute the larger rotation angle to a difference in data, computing method, and the uneven distribution of P-wave first-motion data points in some small earthquakes.

### 3.2. P-Axis and T-Axis Distribution of Focal Mechanism Solution

**Figure 8:**
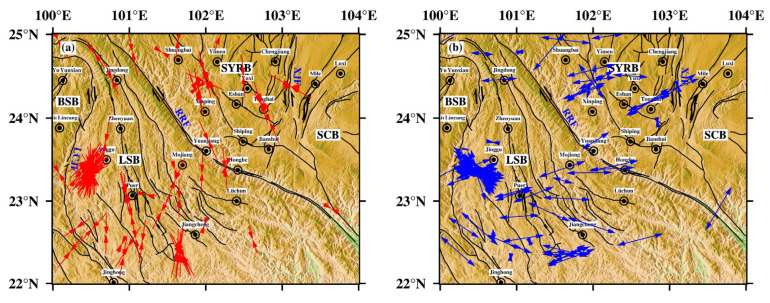
The azimuth distributions of the P-axis (**a**, red arrows) and T-axis (**b,** blue arrows). SYRB: rhombic Sichuan–Yunnan block; LSB: Lamping–Simao block; BSB: Baoshan block; SCB: South China block.

Although the three stress axes of the focal mechanism solution of a single earthquake are not completely consistent with the direction of tectonic stress [40], the dominant direction of the stress axis represents the concentrated distribution characteristics of regional tectonic stress to a certain extent. As shown in Figure 8, we obtained the P-axis and T-axis azimuth distributions of the calculated focal mechanism solutions, and the orientations of the P-axis and T-axis have obvious spatial partitioning characteristics. The P-axis and T-axis of the rhombic Sichuan–Yunnan block have good consistency. The P-axis is distributed in the NNW–SSE direction, and the T-axis is distributed in the ENE–WSW direction. The focal mechanism solutions in the Lamping–Simao block show that the P-axis orientation is in the NNE–SSW direction and the T-axis orientation is in the WNW–ESE direction. The P-axis azimuth changes from NNW–SSE in the northeast of the study area to NNE–SSW in the southwest, and the T-axis azimuth changes from ENE–WSW in the northeast of the study area to WNW–ESE in the southwest, suggesting that the maximum principal stress axis and the minimum stress axis of the study area have clockwise deflection from northeast to southwest.

### 3.3. Stress Variation in the Study Area

To obtain a more comprehensive understanding of the overall characteristics of the tectonic stress field in the study area, we collected other stress data available in the study area for comparative analysis, in addition to calculating part of the focal mechanism data. First, we collected a total of 148 stress data records, including 28 stress relief data records, 26 fault sliding data records, 4 hydraulic fracture data records, and 90 earthquake focal mechanism data records, from the “Basic Crustal Stress Environment Database for Mainland China” [41] and previous studies [42] for the period from 2000 to 2008. In addition, we added focal mechanism solutions of 15 events from July 2019 to April 2021, with data from the Dali Center of the China Earthquake Science Experimental Site. The selected earthquakes range in magnitude from Ms 3.2 to 6.6, with the largest earthquake being the Wuding Ms 6.6 earthquake on 3 June 2007.

**Figure 9:**
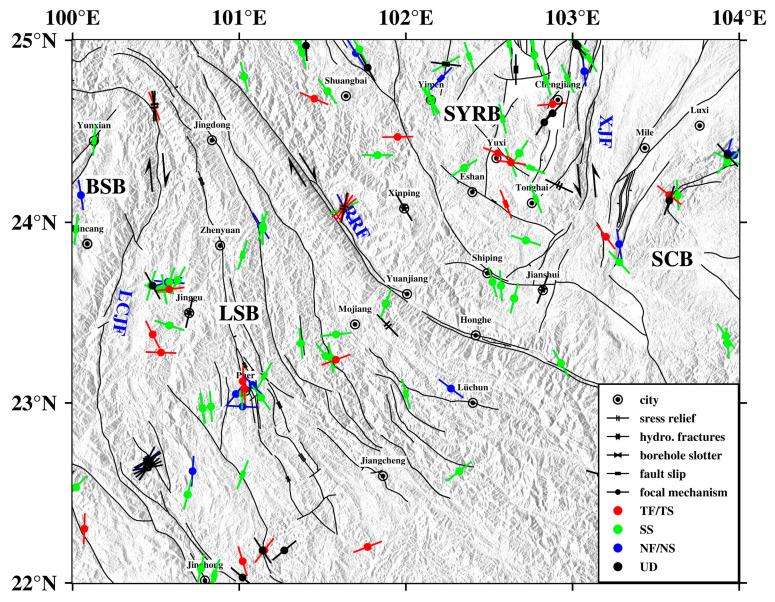
Various types of stress data are available in the study area. NF: normal fault; NS: predominantly normal with strike-slip components; SS: strike-slip; TF: thrust fault (reverse fault); TS: predominantly thrust with strike-slip components; UD: uncertain definition. SYRB: rhombic Sichuan–Yunnan block; LSB: Lamping–Simao block; BSB: Baoshan block; SCB: South China block; LCJF: Lancangjiang (Lancang river) fault; RRF: Red River fault; XJF: Xiaojiang fault.

**Figure 10:**
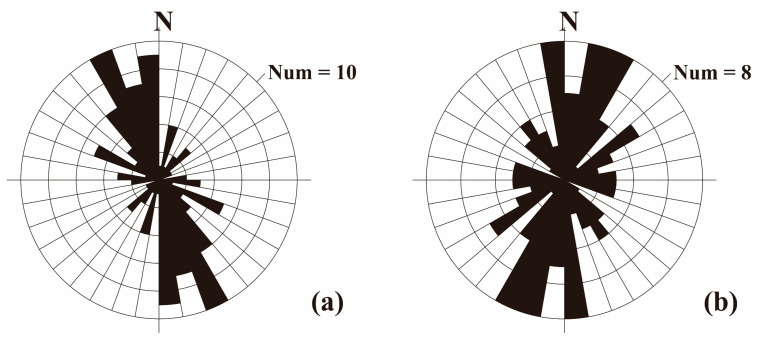
Statistical graph of the azimuth of the maximum principal stress from previous stress data in the study area. (**a**) Northeastern part of the study area. (**b**) Southwest part of the study area.

The P-axis orientation of the focal mechanism solution data and the maximum horizontal principal stress orientation of various types of stress data are shown in Figure 9, indicating that the various types of stress are mainly dominated by the focal mechanism solution data, which are distributed on both sides with the Red River fault as the boundary. The horizontal maximum principal stress direction of the stress data in these two regions was analyzed statistically. The statistical results show that from the Sichuan–Yunnan rhombus in the northeastern part of the southern Yunnan to the Lamping–Simao block in the southwestern part, the maximum horizontal principal stress orientation of all types of stress data generally rotates from the NNW–SSE direction to the NNE–SSW direction (Figure 10), which is consistent with the preliminary understanding obtained from the calculated P-axis distribution of the focal mechanism solution in this study. This also reflects the trend of clockwise rotation of the principal stress orientation of the tectonic stress field in the study area from northeast to southwest.

### 3.4. Stress Field of Each Division Obtained by Grid Inversion

**Figure 11:**
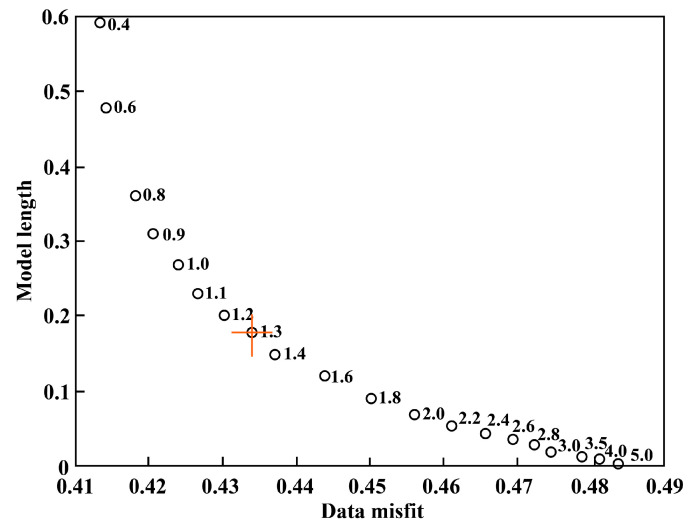
Graph depicting the relationship between the model length and data error. The red cross represents the optimal damping coefficient, and the number on the right of the hollow circle is the corresponding damping.

**Figure 12:**
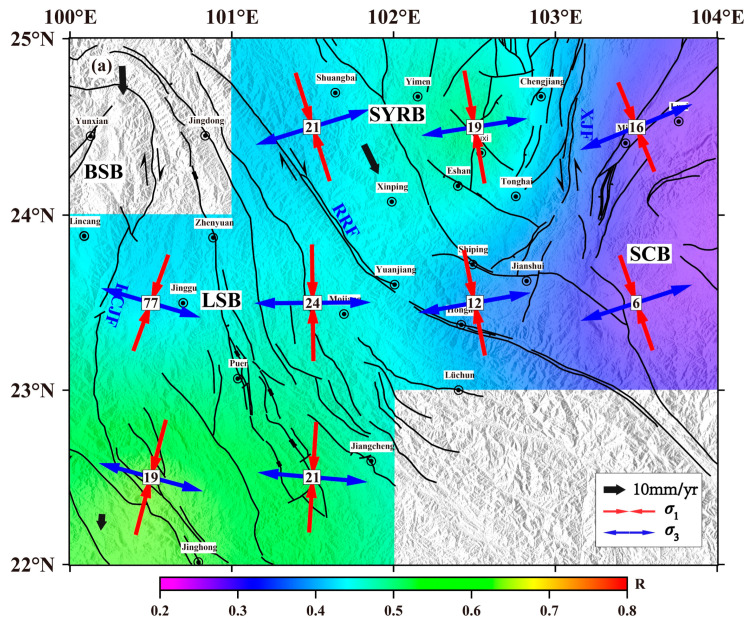

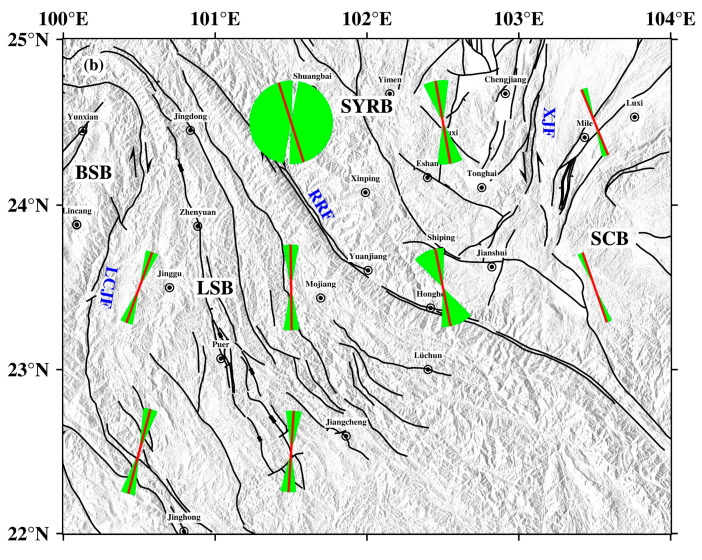
The inversion results and error estimation of the stress field in the study area. (**a**) The distribution of maximum principal stress (*σ*_1_) and minimum principal stress (*σ*_3_). The direction of the line segment represents the orientation of the principal stress, and the length of the line segment represents the magnitude of the inclination angle of the principal stress axis; the longer the line segment is, the smaller the inclination angle is. The number in the small box represents the number of data points involved in the inversion of each grid, and the black thick arrow represents the direction and speed of block movement (modified from Shen et al. [47]). (**b**) Uncertainty estimation of the maximum principal stress azimuth. The green pie chart represents the uncertainty range of the *σ*_1_ axis azimuth obtained from the bootstrap resampling method with 95% confidence intervals, and the red thin line represents the optimal solution of the maximum principal stress.

To further understand the distribution characteristics of the present tectonic stress field in the southern Yunnan region, the study area was divided according to a 1° × 1° grid. Based on the damped region inversion algorithm proposed by Hardebeck et al. [4], the stress field was inverted using the focal mechanism data within each grid. There are 225 focal mechanism solutions involved in the inversion, including 120 event results calculated by using the grid point test method in this study and 105 results collected from other researchers. The number of data points involved in the calculation within each grid was set to at least five, and the specific calculation was completed with the MSATSI program [43]. The optimal damping coefficient selected in this paper was 1.3 (Figure 11), the confidence interval of the inversion results was 95%, and the bootstrap resampling times were 2000. The inversion results (Figure 12a, Table 2) show that the maximum principal stress axis (*σ*_1_ axis) and minimum principal stress axis (*σ*_3_ axis) rotate clockwise from northeast to southwest, and the *σ*_1_ axis presents a radial distribution, which is consistent with previous results [13,23,44,45]. The *σ*_1_ axis of the rhombic Sichuan–Yunnan block and South China block located in the northeastern part of the study area is NNW–SSE, and the azimuth ranges from 157.88° to 169.83°. The *σ*_3_ axis is ENE–WSW, and the azimuth ranges from 67.70° to 80.87°. In the Lamping–Simao block located in the southwest, the *σ*_1_ axis of the block is rotated in the NNE–SSW direction with an azimuth range of 3.68~179.27°, while the *σ*_3_ axis is in the WNW–ESE direction with an azimuth range of 89.55~94.97°. The maximum clockwise rotations of the *σ*_1_ axis and the *σ*_3_ axis from the northeast end to the southwest end are 36.85° and 37.48°, respectively, which are obviously larger than the uncertainty range of the inversion results of each grid point (essentially within 20°). Meanwhile, the dip angles of the *σ*_1_ and *σ*_3_ axes are very small and close to horizontal, showing the strike-slip stress regime. The main fault structures in southern Yunnan are mainly strike-slip types [46], which reflects that the tectonic activities in this area are mainly controlled by the regional stress field, and the seismic activities are mostly manifested by strike-slip focal mechanisms (approximately 50% of the total). The distribution of the *R*-values of the rhombic Sichuan–Yunnan block and the South China block north of the Red River fault gradually decreases from northwest to southeast. The larger *R*-value located in the northwest shows the characteristics of tensile stress, while the smaller R-value in the southeast shows the characteristics of compressive stress. Additionally, the distribution of the *R*-value in the Lamping–Simao block south of the Red River fault shows a trend of increasing gradually from north to south. To the north of the small *R*-value, the intermediate principal stress axis shows compressive stress characteristics, while to the south of the large *R*-value, the central principal stress axis shows tensile stress characteristics.

Moreover, from the uncertainty range of the maximum principal stress azimuth with 95% confidence intervals (Figure 12b), the inversion results of most grid points in the study area vary within 20°, indicating that the inversion results are relatively stable. However, the inversion results vary widely near the Red River fault and the intersection area of the Red River fault and Xiaojiang fault, suggesting a relatively low uniformity degree and data fitting degree of the tectonic stress field in these areas, which has the characteristics of nonuniformity.

## 4. Discussion

The southern Yunnan is located at the junction of the Eurasian plate and the Indian plate, which is not only affected by the extrusion force eastward in the main area of the Tibetan Plateau but also influenced by the deep subduction of the Burma arc, the wedging of the eastern Himalaya structure, and the self-gravitational collapse of various forces [48]. In addition, the southern Yunnan was blocked by the rigid blocks of the Sichuan Basin and South China in the east, causing a complex geodynamic environment and strong tectonic activity in this area [49]. These dynamic effects promoted the development of late Quaternary active faults, which divided the boundary of the study area into several blocks. The movement modes and speeds of different active blocks were different, and the differential movement between blocks was strongest at their boundary [50]. According to the distribution of the tectonic stress field (Figure 12), the variation in the direction of maximum principal stress also clearly verifies the differential characteristics of block motion. The rhombic Sichuan–Yunnan block is located on the channel where the material of the Qinghai-Tibet Plateau is prevented from escaping eastward and migrating southward. The inner part of the block exhibits overall NNW–SSE compression. Due to the obstruction of the South China block, the maximum principal stress in the southeastern boundary zone of the rhombic Sichuan–Yunnan block has a certain degree of clockwise rotation compared with the inner part of the block. The Lamping–Simao block is adjacent to the Burma arc plate subduction zone to the west, and the Indian plate is obviously subducted under the Burma arc mountains [51]. The NE migration of the Indian plate and the eastward extrusion of the Burma arc play a dominant role, and the maximum principal stress direction of the western region of the block is mainly the NNE–SSW direction. In the inner Lamping–Simao block, due to the proximity of the Sichuan–Yunnan rhomboid-shaped block and Baoshan block on the east and west sides, the direction of maximum principal stress is mainly near the NS direction under the joint action of the NE wedge of the East Himalayan tectonic junction and SE extrusion of the Qinghai–Tibet Plateau.

The stress distribution in the block is relatively isostatic; however, there are certain changes in the stress orientation among different blocks. Generally, the orientation of the maximum principal stress *σ*1 in the study area shows a clockwise rotation trend from the northeast to the southwest. GPS horizontal velocity field results and other stress data, such as in situ stress measurements, also indicate that the regional deformation and horizontal stress distribution in the study area have obvious clockwise deflection [51,52]. Meanwhile, the observations of the GPS surface velocity field correspond with the results of the tectonic stress field. In particular, the direction of the maximum principal stress inside the block is basically parallel to the direction of the GPS horizontal velocity field. The tectonic stress field inverted by focal mechanisms (mainly distributed at depths of 5–20 km, Figure 4b) reveals the stress state in the upper crust, while the results of GPS and in situ stress measurements represent the deformation and stress distribution of the earth’s surface. These stress–strain data reflecting different depths of the crust show good consistency, indicating that the different blocks in the study area are dominated by integral deformation and movement, and the movement modes of the deep and shallow parts are similar. Additionally, the stress distribution in the block boundary zone is more complicated in the adjacent parts of the block, which may be related to the different movement modes and speeds of the secondary blocks and boundary fault zones.

In terms of the distribution of the *R*-value of the stress shape factor, the *R*-value of the Lamping–Simao block generally increases from north to south, indicating that the intermediate principal stress is mainly manifested as the characteristics of tensile stress, and the relative proportion of compressive stress in the whole stress decreases. It can be inferred that the weakening of extrusion will make the movement velocity of the block gradually decrease from north to south. Gan et al. [53] found that the GPS horizontal velocity in the southeastern margin of the Tibetan Plateau showed a gradually decreasing trend from north to south, suggesting that the gradual slowing of underground material escape from north to south was consistent with GPS observations and manifested through the background tectonic stress field. The reason for the slow movement of the blocks in the study area may be that during the eastward escape of the Tibetan Plateau material, its eastward slip movement was blocked by the eastern South China block and forced it to deflect southward, and the speed decreased accordingly. Meanwhile, the Lamping–Simao block and the southern Baoshan block were far away from the strong compressive area of the East Himalaya structure, and the motion velocity and displacement of the block are smaller than those in the north, which is essentially consistent with the results of the block motion velocity calculated by Shen et al. [47] from GPS data. Their research also found that the rotation rate of the block also has a decreasing trend from north to south. In our study, the *R*-value distribution of the South China block is significantly smaller than that of the inner Sichuan–Yunnan block, indicating that the intermediate principal stress is mainly characterized by compressive stress, and the relative proportion of compressive stress in the whole stress increases, indicating that the compression action in this region is stronger, which may be related to the strong resistance of the rhombic Sichuan–Yunnan block to the east of the South China block.

In addition, according to the simulation of the focal mechanism under the strike-slip stress system from Wan [54], the number of normal fault type and normal strike-slip type focal mechanism solutions will gradually decrease when the *R*-value increases, while the proportion of reverse fault and reverse strike-slip focal mechanisms in all earthquakes will gradually increase. From the distribution of the focal mechanism of earthquakes in the study area, compared with the area with a larger *R*-value, there are more normal fault and normal strike-slip events in the area with a smaller *R*-value, and fewer reverse fault and reverse strike-slip events, which is essentially consistent with the simulation results. Therefore, it is of reference significance to analyze the relationship between the tectonic stress field and earthquakes and to explain the geodynamic process when studying the tectonic stress field combined with the *R*-value.

The influence of large earthquakes (e.g., the 2008 Mw 8.0 Wenchuan earthquake) nearby on the tectonic stress field in Southern Yunnan is an issue worth further discussion. He et al. [55] suggested that the background tectonic stress field is relatively stable and that even a large earthquake can only change the background tectonic stress field in a small range near the earthquake source but cannot change the general trend distribution of the tectonic stress field. The effect of the Wenchuan earthquake on the tectonic stress field in southern Yunnan may need to be further explored with more geological and geophysical data validation.

## 5. Conclusions

In this study, the focal mechanism solutions of some *M*_L_ ≥ 2.5 seismic events in the southern Yunnan region were calculated by using the modified grid point test method, focal mechanism solutions of historical earthquakes were collected, and the regional tectonic stress field of the study area was calculated with the inversion method. Combined with other stress data, we studied and analyzed the variation characteristics of the tectonic stress field in the study area, its relationship with earthquakes, and its dynamic significance. The following conclusions and understandings can be obtained:

(1) The focal mechanism solution types of earthquakes in the southern Yunnan region are complex and diverse, with the main type of earthquakes being strike-slip events, followed by normal fault and reverse fault events.

(2) The stress field inversion results show that the maximum and minimum principal stress axes rotate clockwise from northeast to southwest in southern Yunnan, and the direction distribution of the maximum principal stress axis is similar to the GPS horizontal velocity field and other stress data. The direction of the maximum principal stress inside the block is consistent, and there are some changes in the distribution of the stress field between the block boundaries and different blocks, which may be due to the difference in the dynamic action, movement mode, and velocity of the block.

(3) The collision between the Eurasian plate and the Indian plate caused the continuous eastward extrusion of the Tibetan Plateau material. The maximum principal stress of the Sichuan–Yunnan block, which is far away from the collision boundary of the two plates, is mainly controlled by the southeast force action of the Tibetan Plateau, and the inner part of the block exhibits overall NNW–SSE compression. In the Lamping–Simao block, which is adjacent to the Burma arc plate subduction zone to the west, the NE migration of the Indian plate and the eastward extrusion of the Burma arc play a dominant role, and the maximum principal stress direction of the western region of the block is mainly the NNE–SSW direction.

(4) The distribution of the *R*-value of the stress shape factor in the Lamping–Simao block gradually increases from north to south in general, and the relative proportion of compressive stress in the whole stress decreases, resulting in a decrease in the migration speed and displacement of the block, which is consistent with the observed surface deformation results. The *R*-value distribution of the South China block is significantly smaller than that of the rhombic Sichuan–Yunnan block, and the proportion of compressive stress is larger, indicating a stronger extrusion in the region, which may be related to the strong obstruction of the rhombic Sichuan–Yunnan block by the eastern South China block. The analysis of the tectonic stress field combined with the *R*-value has certain reference significance for explaining the geodynamic process in the study area.

## Figures and Tables

**Table 1:** Classification standard of focal mechanism solutions.

Regime	Plunge of *P* Axis	Plunge of *B* Axis	Plunge of *T* Axis
NF	≥52°		≤35°
NS	40° ≤ plunge ≤ 52°		≤20°
SS	≤20° <40°	≥45° ≥45°	<40° ≤20°
TS	≤20°		40° ≤ plunge ≤ 52°
TF	≤35°		≥52°
UD	None of the above types		

**Table 2:** The stress field parameters of each division obtained via grid inversion.

Grid Coordinates		*σ*_1_ Axis		*σ*_2_ Axis		*σ*_3_ Axis		*R* Values
Lon/°	Lat/°	Azimuth/°	Plunge/°	Azimuth/°	Plunge/°	Azimuth/°	Plunge/°	
100.5	22.5	14.73 5.6~24.4	2.42 −44.6~12.8	−88.18 −265.3~89.5	79.30 51.8~89.5	105.18 96.5~113.0	10.41 −19.5~38.2	0.62 0.34~0.93
100.5	23.5	−159.99 −169.7~−150.3	14.43 −1.4~42.6	−24.85 −82.5~23.7	70.05 53.8~82.3	106.48 97.1~115.2	13.48 −0.4~28.9	0.42 0.17~0.69
101.5	22.5	3.68 −7.0~15.1	8.51 −45.0~23.3	−130.56 −309.8~48.6	77.90 52.3~89.9	94.97 83.7~105.6	8.54 −21.2~36.6	0.55 0.23~0.89
101.5	23.5	−0.73 −10.9~10.0	3.94 −45.0~34.7	−134.81 −314.8~44.9	84.34 54.8~89.8	89.55 0.0~99.9	4.05 −16.9~24.7	0.46 0.06~0.82
101.5	24.5	162.47 10.4~182.1	6.29 −65.6~88.0	0.71 −178.8~180.5	83.38 −5.3~89.8	−107.30 −123.4~−92.6	2.05 −17.6~ 33.3	0.41 0.02~0.87
102.5	23.5	−11.24 −44.9~1.5	10.70 −59.0~77.5	−161.56 −340.9~18.4	77.73 9.5~89.7	79.89 65.8~91.9	5.94 −22.3~22.0	0.37 0.02~0.76
102.5	24.5	169.83 152.8~187.2	5.47 −26.5~ 44.9	53.30 −125.8~231.9	77.90 6.2~89.9	−99.13 −115.7~−53.6	10.76 −60.6~81.5	0.53 0.01~0.98
103.5	23.5	−19.00 −197.8~152.7	15.88 −74.0~90.0	174.45 −5.4~354.2	73.70 −15.9~89.5	72.02 60.8~86.9	3.6 −16.5~17.8	0.28 0.01~0.72
103.5	24.5	157.88 −12.2~335.9	18.97 −67.7~84.9	−23.84 −203.8~156.1	71.20 −11.2~89.4	67.70 50.9~86.6	0.53 −25.6~27.4	0.29 0.00~0.82

Note: the uncertainty range of parameter is under 95% confidence.

## Data Availability

All data are available in the main text.
